# Editorial for the Special Issue on Micro-Machining: Challenges and Opportunities

**DOI:** 10.3390/mi9110564

**Published:** 2018-10-31

**Authors:** Xichun Luo, Wenlong Chang, Jining Sun

**Affiliations:** 1 Centre for Precision Manufacturing, Department of Design, Manufacture and Engineering Management, University of Strathclyde, Glasgow G1 1XJ, UK; wenlong.chang@strath.ac.uk; 2 Insitute of Mechanical, Process and Energy Engineering, School of Engineering and Physical Science, Heriot Wat University, Edinburgh EH14 4AS, UK; Jining.Sun@hw.ac.uk

Micro-machining is an enabling technology for the manufacture of micro-products in which functional features, or at least one dimension, are in the order of μm. This is pivotal to our economy, as micro-products, such as micro displays, micro batteries, and micro fluidics, etc. are becoming well-established in all major areas of our daily life and can already be found across a broad spectrum of applications, especially in the automotive, aerospace, photonics, renewable energy, and medical instrument sectors.

Nowadays micro-machining technologies are clearly advancing towards the economical manufacturing of customized high precision 3D micro-products made of a variety of materials, including difficult-to-machine materials such as glass, sapphire, ceramics, and hard steels, etc. This proposes significant research challenges in terms of process control, but also provides great opportunities for the research and development new advanced micromachining technologies in areas such as modelling, hybrid micro-machining, and in-line/on-machine metrology, to name a few.

This special issue of *Micromachines*, entitled “*Micro-Machining: Challenges and Opportunities*”, collects 16 original research papers and one review article that provides updates on the latest micro-machining technologies, including novel research on and development of diamond turning [1,2], micro-milling [3,4,5], micro-grinding [6], polishing [7,8,9], laser micro-machining [10,11,12], lithography-based micro-machining processes [13], ultrasonic devices [14], control systems for hybrid machines [15], on-machine surface metrology [16], and the surface and subsurface integrity characterization technique [17].

Adopting difficult-to-machine materials for emerging 3D micro-products proposes a great challenge to micro-machining technology. The so-called “size effect” material removal mechanism in micro-machining is different from that of conventional machining. Li et al. [1] applied molecular dynamics simulations to gain an in-depth understanding of formation mechanism of surface roughness and residual stress of single crystal cerium in ultra-precision diamond turning process. Their studies revealed that dislocation and lattice distortion were two factors that govern the formation of surface roughness and residual stress. This finding has been used to optimize machining parameters to achieve desired surface roughness and residual stress. Tungsten carbide is another difficult-to-machine material used for optical lens moulds. Research by Li, Fang and Jia [2] showed that abrasive tool wear and micro chipping were major tool wear mechanisms in diamond turning of tungsten carbide. They researched and developed a novel ultrasonic assisted diamond turning device to restrain tool wear and obtain nano-smoothed surface. To further extend the application of ultrasonic technology, Fang et al. [14] designed a novel ultrasonic transducer based on rare-earth giant magnetostrictive materials which can overcome problems of small vibration amplitude and power loss for current piezoelectric ceramic transducers. They applied this new transducer in a surface strengthening experiment for #40 steel and demonstrated a mirror surface finish and 20% increase of surface hardness of the test part.

As the most extensively developed micro-machining technology, micro-milling provides a great degree of high machining efficiency and flexibility for 3D micro-products, moulds, and dies. Due to the use of small diameter milling cutter surface uniformity and tool wear becomes two major research problems in micro-milling. Sun et al. [3] thoroughly investigated the influence of periodically variation of chip thickness and ploughing effect on surface uniformity and proposed a mathematical prediction model. Their study showed that surface uniformity deteriorated with the increase of feed rate increases and blunt of cutting edge. However, increase of depth of cut could improve surface uniformity. ZrO_2_ is the second most-used ceramic on the market. It has been extensively used for impellers for turbo-machinery, diesel injection micro nozzles, micro-fluidic devices, micro-moulds, oxygen sensors for foundry industry, dental and orthopaedic implants, etc. However, it is a difficult-to-machine material due to its high hardness and brittleness. Bian et al. [4] has successfully developed a ductile regime micro-milling process for ZrO_2_. Mirror surface quality with a surface roughness (Ra) of 0.02 μm was achieved through the development. Micro-milling mould and dies is an important manufacturing step in the process chain for mass production of micro-products such as micro-lens and microfluidics. Its attainable machined surface and accuracy will significantly influence the performance of formed/replicated micro-products. Gao et al. [5] proposed a novel 3D offset spiral strategy which helped achieve good surface finish and machining accuracy on AISI H13 steel micro-lens array mould.

SiC is regarded as one of the most difficult-to-machine materials due to its extremely high hardness and brittleness. Micro-grinding is commonly used as the first processing step for SiC. In order to control and optimize the grinding process, Li et al. [6] proposed a new comprehensive grinding force model to predict the force components which took into account of material brittle fracture, grinding conditions and random distribution of the grinding wheel topography. The evaluation trial had approved the effectiveness of this novel grinding force model.

In recent years, hybrid micro-machining technology has emerged as a new solution for the economical manufacturing of high precision 3D micro-products made of hard-to-machine materials. As it can offer a great benefit to obtain the so-called “1 + 1 = 3” effect, hybrid micro-machining has been applied to all major micro-machining processes included polishing and finish processes. Lin et al. [7] proposed a novel disc hydrodynamic polishing (DHDP) process through combining elastic emission machining and fluid jet polishing. The evaluation experiment had demonstrated the new hybrid polishing process could produce ultra-smooth surfaces (surface roughness Ra of 2 nm) efficiently without subsurface damage and surface scratches on fused quartz glass. Guo and Suziki [8] thoroughly studied the influence of process parameters such as vibrating motion, abrasives, pressure and tool wear on material removal rate and surface roughness in vibration-assisted polishing proves for micro-optic mould. Their experimental results showed that 2D vibrating motion generated better surface roughness with higher material removal efficiency while a smaller grain size of abrasives created better surface roughness but lower material removal efficiency. Guo et al. [9] performed a systematic study of the effect of processing parameter on surface integrity in magnetic field-assisted finishing (MFAF) of RSA 905 which is a challenging material for normal polishing process. A high degree material removal and low surface roughness were obtained in the experimental trials. The research showed that MFAF could help release residual stress and improve tribological performance of the polished part.

Hybrid micro-machines are platforms to accommodate hybrid machining processes through integration of multiple functional modules from different vendors for the best value and performance. Currently, the lack of plug-and-play solutions leads to tremendous difficulty in system integration. Luo, Zhong and Chang [15] proposed a novel three-layer control architecture, for the first time, for the system integration of hybrid micromachines. They verified the effectiveness of the proposed control architecture through the integration of a six-axis hybrid micro-machine. This new control architecture provides invaluable guidelines for the development of next-generation CNC systems for hybrid micro-machines.

Laser micro-machining uses laser beam to remove material through ablation. It is a very effective manufacturing approach to generate microstructures on hard-to-machine materials due to no concern of tool wear. Mohammed et al. [10] applied an Nd: YAG laser to direct write microchannels on a hard-to-machine material—aluminium ceramic used for microreactors, microfluidic devices and microchemical systems. Their research showed that beam intensity has a major influence on dimensional accuracy and material removal rate. Low intensity and low to medium pulse overlap could result in good dimensional accuracy. In recent years laser micromachining has been widely used to generate functional microstructured surfaces for medical and chemical analysis applications. Kim and Noh [11] used CHF_3_ plasma etching to generate hydrophobic nanospikes surfaces and laser ablation to generate hydrophilic channel on its top which can be used for cell growth, protein manipulation, the spotting of biomolecules, micro-fluidics and water collection. Their research showed that laser heat affected zone could be reduced with the decrease the width of hydrophilic channels. This novel functional surface could be used for electric flexible circuit. Cruz-Ramirez et al. [12] developed a novel system and process for local laser microablation or polymerization for true on-demand biomimetic micropatterned designs in transparent polymers and hydrogels. This novel development allowed integration of microfluidics, microelectronics, surface microstructuring, and transfer of superficial protein micropatterns on a variety of biocompatible materials.

Lithography base micro-machining is also an important fabrication approach for micro-components and micro-parts. Cao et al. [13] proposed a new method for the fabrication of microwell arrays with randomly varied diameters as new structural elements for pseudo-thermal speckle light source for quantum imaging application. This novel method is simple and can be easily scaled up for large structures.

On-machine surface measurement is currently a research focus in micro-machining as it can avoid the re-positioning error of off-line measurement and provide immediate feedback to machining operation for higher quality control. Li et al. [16] proposed a systematic methodology for kinematics error modelling, measurement, and compensation of an on-machine surface measurement system installed on an ultra-precision turning machine. The research showed that the result of on-machine surface measurement is the superposition of the sample surface form error and the machine tool kinematics error. When applying the proposed error compensation approach, the accuracy of the characterized flatness error obtained from the on-machine surface measurement system can be improved by 67%.

Surface and subsurface integrity characterisation is a very important task in micro-machining as it will directly influence the performance of micro-parts. Xu et al. [17] performed a critical review of application of Raman spectroscopy as an efficient, powerful, and non-destructive testing method to characterize microstructures changes, surface and subsurface damages and residual stress induced by micro-machining. The Tip-Enhanced Raman Spectroscopy (TERS) technique which can dramatically enhance the Raman scattering signal at weak damages has been considered as a promising research field in this review article.

We would like to take this opportunity to thank all the contributing authors for their great efforts to presenting their excellent research work to this special issue. Our appreciation also goes to all the reviewing experts who have provided timely invaluable comments and suggestions for the further improvement of the quality of the research papers. The tremendous support from the editorial staff of *Micromachines* is also highly appreciated.
